# Dihydrocapsaicin down-regulates apoM expression through inhibiting Foxa2 expression and enhancing LXRα expression in HepG2 cells

**DOI:** 10.1186/1476-511X-13-50

**Published:** 2014-03-19

**Authors:** Jia-Yi Zhao, Yan-Wei Hu, Shu-Fen Li, Ya-Rong Hu, Xin Ma, Shao-Guo Wu, Yan-Chao Wang, Ji-Juan Gao, Yan-Hua Sha, Lei Zheng, Qian Wang

**Affiliations:** 1 Laboratory Medicine Center, Nanfang Hospital, Southern Medical University, Guangzhou, Guangdong 510515, China; 2 Department of Anesthesiology, Nanfang Hospital, Southern Medical University, Guangzhou, Guangdong 510515, China

**Keywords:** DHC, ApoM, Foxa2, LXRα

## Abstract

**Background:**

Apolipoprotein M (apoM), as a novel apolipoprotein which is mainly expressed in liver and kidney tissues, is associated with development and progression of atherosclerosis and diabetes. Our group have recently shown that Dihydrocapsaicin(DHC)can significantly decrease atherosclerotic plaque formation in apoE−/− mice. However, the effect and possible mechanism of DHC on apoM expression remain unclear.

**Methods:**

HepG2 cells were treated with 0 μM, 25 μM, 50 μM and 100 μM DHC for 24 h or were treated with 100 μM DHC for 0, 6, 12, and 24 h, respectively. The mRNA levels and protein levels were measured by real-time quantitative PCR and western blot analysis, respectively.

**Results:**

We found that DHC markedly decreased expression of apoM at both mRNA and protein level in HepG2 cells in a dose-dependent and time-dependent manner. Expression of Foxa2 was decreased while expression of LXRα was increased by DHC treatment in HepG2 cells. In addittion, overexpression of Foxa2 markedly compensated the inhibition effect induced by DHC on apoM expression. LXRα small interfering RNA significantly abolished the inhibition effect which induced by DHC on apoM expression. The liver of C57BL/6 mice treated with DHC had significantly lower expression of apoM. Furthermore, the liver had lower expression of Foxa2 while had higher expression of LXRα.

**Conclusions:**

DHC could down-regulate apoM expression through inhibiting Foxa2 expression and enhancing LXRα expression in HepG2 cells.

## Introduction

Capsaicinoids, including capsaicin and dihydrocapsaicin(DHC), which together typically represent 85–90% of the total capsaicinoid content in ‘hot chilli peppers’ extract, and its minor components include nordihydrocapsaicin, homocapsaicin and homodihydrocapsaicin [1,2]. These spice principles have been proved to augment carbohydrate metabolism [3,4], energy expenditure [5] and lipid metabolism [6-8] in rodents and/or humans, and have been successfully used in the treatment of diverse clinical conditions such as relief the pain of peripheral neuropathies, sympomatic treatment of rheumatoid arthritis and osteoarthritis [9]. Previous studies have demonstrated that regular consumption of chilli increases the resistance of oxidation of serum lipoproteins *in vitro*[6] and attenuate postprandial hyperinsulinemia [10], actions that may help in reducing cardiovascular diseases (CVD) risk. Adams et al. have demonstrated that capsaicin and DHC could inhibit *in vitro* platelet aggregation and the activity of clotting factors VIII and IX, a property which may contribute to the prevention of the onset and/or treatment of CVD [11]. Furthermore, our group have recently shown that DHC can significantly decrease atherosclerotic plaque formation involving in a PPARγ/LXRα pathway in apoE−/− mice fed a high-fat/high-cholesterol die [1]. These reports support the notion that capsaicinoids associate with CVD, such as atherosclerosis and coronary heart disease in particular.

Apolipoprotein M (apoM) was first described by Xu and Dahlbäck in 1999 [12]. ApoM is a member of the lipocalin protein superfamily, whose members exhibit diverse properties such as lipid binding, transport, and immunological functions [13,14]. ApoM, mainly expressed in hepatocytes and in the tubular epithelial cells of the kidney, is mainly associated to HDL (96% is bound to HDL), but also binds to low density lipoprotein (LDL), very low density lipoprotein (VLDL) and chylomicrons [12,15-17]. It has been proved that apoM plays an important role in formation of pre-β-HDL and cholesterol efflux to HDL, which further influences the HDL cholesterol concentration in plasma. Moreover, the silencing of apoM expression was associated with the absence of pre-β-HDL particles in plasma [18]. In addition, plasma apoM is modestly reduced in patients with diabetes compared to controls [19]. Futhermore, Serum apoM concentrations and hepatic *APOM* mRNA levels were significantly reduced in the hyperglycemic rats, indicating that the low expression levels of apoM in these diabetic animals could be ascribed to hyperglycemia [20]. These observations support the notion that apoM is linked to cholesterol metabolism and diabetes.

FOXA genes, formerly termed HNF3 (hepatocyte nuclear factors), is transcription factor involved in glucose homeostasis and lipid metabolism in liver [21,22]. Foxa2 is phosphorylated and excluded from the nucleus when plasma insulin levels increase [23]. A binding site for Foxa2 in the *APOM* promoter is at position −474. It had been proved that obese mice had decreased apoM expression and plasma pre-β-HDL levels due to inactivation of Foxa2 in the hyperinsulinemic state. Treatment wild-type mice and ob/ob mice with an adenovirus containing phosphorylation-defective Foxa2 not only improved glucose and lipid homeostasis but also increased hepatic apoM mRNA expression. In contrast, haploinsufficient Foxa2+/−mice exhibited decreases in hepatic apoM expression and in plasma pre-β-HDL and HDL levels [24]. Together, these results suggest that Foxa2 regulates *APOM* transcription.

Liver X Receptor α (LXRα) is a major transcriptional regulator of cholesterol homeostasis and also regulates lipid and glucose metabolism [25,26]. LXRα is more restricted and mainly found in liver, intestine, fat tissue,macrophages, kidney and gonads, suggesting their important function in the control of cholesterol homeostasis, whereas LXRβ is expressed in most cell types [27]. Zhang et al. demonstrated that LXR agonist, TO901317,could decrease hepatic apoM expression in the vivo and *in vitro*. They showed that serum apoM of mice treated with 100 mg/kg/d of TO901317 decreased significantly as compared to control mice. In cultured HepG2 cells, TO901317 caused a downregulation of apoM expression, indicating that apoM is another target gene of LXR. The combination of 9-cis-retinoic acid (RA) and T0901317 showed additive effects, which suggests that apoM expression is modulated by the LXR/RXR pathway [28].

However, the relation between the DHC and apoM in HepG2 cells remain unclear. whether Foxa2 and LXRα-dependent pathway involving in this progress has not yet been explored. In the present study, we demonstrated that DHC could markedly down-regulate apoM expression through inhibiting Foxa2 expression and enhancing LXRα expression in HepG2 cells.

## Materials and methods

### Materials

Dihydrocapsaicin (N-[(4-hydroxy-3-methoxyphenyl) methyl]-8-methyl-6-nonanamide) was purchased from Sigma Chemical Company (St. Louis, MO, USA). The PrimeScript RT Reagent Kit (Perfect Real Time) (DRR037A) (TaKaRa, Japan), SYBR Premix Ex TaqTM II (Tli RNaseH Plus) (DRR820A) (TaKaRa,Japan) were obtained as indicated. All other chemicals were of the best grade available from commercial sources.

### Animals

Eight-week-old, female C57BL/6 mice (Laboratory Animal Center of Peking University, Beijing, China) with a mean body mass of 20 g were randomly divided into two groups(n = 5/group): (1) Control group were treated with cholesterol-free vegetable oil by oral gavage (0.2 mL per mouse) for one week. (2) DHC group were treated with cholesterol-free vegetable oil containing DHC (3.0 mg/kg body weight) daily by oral gavage(0.2 mL per mouse) for one week. All animals were housed five per cage at 25°C on a 12-h/12-h light/dark cycle with unlimited access to chow and water. The animal procedures were approved by the Animal Experimental Committee at Nanfang Hospital (Guangdong, China).

### Cell culture

Human hepatocytes (HepG2) was purchased from the American Type Culture Collection(ATCC, Manassas, VA, USA). The HepG2 cells were grown in Dulbecco’s modified Eagle’s medium (DMEM) supplemented with 10% fetal calf serum (FCS) and 1% penicillin/streptomycin. Cells were incubated at 37°C in an atmosphere of 5% CO2. Cells were seeded in 6-or12-well plates or 60-mm dishes and grown to 60%–80% confluence before use.

### Chemicals

DHC was initially dissolved in 100% ethanol to make a stock solution of 1 mol/L and then diluted for further experiments in normal buffered saline. The ethanol concentration of the dilutions was 0.1%. Normal buffered saline containing 0.1% ethanol was used as the solvent in the control cells. Dilutions were prepared from stock solutions shortly before the experiment.

### RNA isolation and real-time quantitative PCR analysis (qPCR)

Total RNA from mouse tissues or cultured cells was extracted using TRIzol reagent (Invitrogen) according to the manufacturer’s instructions. Real-time quantitative PCR, using SYBR Green detection chemistry, was performed on an ABI 7500 Fast Real Time PCR system (Applied Biosystems, Foster City, CA, USA). The following primers were used: *apoM* forward, 5′-CTGAATGAGACAGGCCAGGGTTA-3′; *apoM* reverse, 5′-CAGGTCAGTTATTGGACAG CTCACA-3′; *Foxa2* forward, 5′-CGTCCGACTGGAGCAGCTACTAT-3′; *Foxa2* reverse, 5′-AT GTACGTGTTCATGCCGTTCA-3′; *LXRα* forward, 5′-TCTGGAGACATCTCGGAGGTAC AAC-3′; *LXRα* reverse, 5′-AGCAAGGCAAACTCGGCATC-3′; *GAPDH* forward, 5′-GACT CATGACCACAGTCCATGC-3′; *GAPDH* reverse, 3′-AGAGGCAGGGATGATGTTCTG-5′. Melt curve analyses of all real-time PCR products were performed and shown to produce a single DNA duplex. All samples were measured in triplicate and the mean value was considered for comparative analysis. Quantitative measurements were determined using the ^△△^Ct method and GAPDH expression was used as the internal control.

### Western blot analyses

Proteins were extracted from mouse tissues or cultured cells using RIPA buffer (Biocolor Ltd., Belfast, Northern Ireland, UK), quantified using the BCA protein assay kit (KeyGen Biotechnologies, Nanjing, China), and then subjected to western blot analyses (10% sodium dodecyl sulfate–polyacrylamide gel electrophoresis; 30 μg protein per lane) using rabbit polyclonal anti-APOM antibodies (BD Bio-sciences, San Jose, CA, USA), rabbit polyclonal anti-Foxa2 antibodies (Epitomics., CA, USA), and rabbit polyclonal anti-LXRα (Proteintech group, Inc., Chicago, IL, USA) and β-actin-specific antibodies (Abcam Inc.,Cambridge, MA, USA). The proteins were visualized using a chemiluminescence method (ECL Plus Western Blot Detection System; Amerisham Biosciences, Foster City, CA, USA).

### Transfection with small interfering RNA (siRNA)

The siRNAs against Foxa2 and LXRα and an irrelevant 21-nucleotide control siRNA (Negative Control) were purchased from Ribo Biotechnology. Cells (2 × 10^6^ cells/well) were transfected using Lipofectamine2000 transfection reagent for 48 h according to the manufacturer’s instructions. After 48 h of transfection, real-time RT-PCR and western blotting were performed.

### Construction of recombinant plasmids

The PIRES2-EGFP and PCR-XL-TOPO vectors (containing Foxa2 or LXRα which was assembled by the chemically synthesized oligos through PCR) were purchased from Invitrogen. Segments of EcoRI-Foxa2 or LXRα and IRES-EGFP-XhoI were amplified using the template of the PCR-XL-TOPO and PIRES2-EGFP vectors, respectively. EcoRI-Foxa2-IRES-EGFP-XhoI or EcoRI- LXRα-IRES-EGFP-XhoI was joined by the two above-mentioned segments using overlap PCR. Gel electrophoresis was performed and the relevant band was excised from the gel, double enzyme-digested by EcoRI/XhoI, incorporated into the pcDNA3.1(+) vector, and then transformed into competent *E.coli* DH5a cells for further amplification and use. The recombinant plasmids were verified by sequencing and named pcDNA3.1-Foxa2 and pcDNA3.1-LXRα. The plasmid transfection process was performed using Lipofectamine 2000 transfection reagent according to the manufacturer’s instructions.

### Statistical analyses

Data are reported as means ± S.D. The date were compared by one-way analysis of variance and the Student’s *t*-test, using the Statistical Package for the Social Sciences (version13.0) software (SPSS, Inc., Chicago, IL, USA). Statistical significance was obtained when *p* values were less than 0.05.

## Results

### DHC down-regulates apoM expression in HepG2 cells

**Figure 1:**
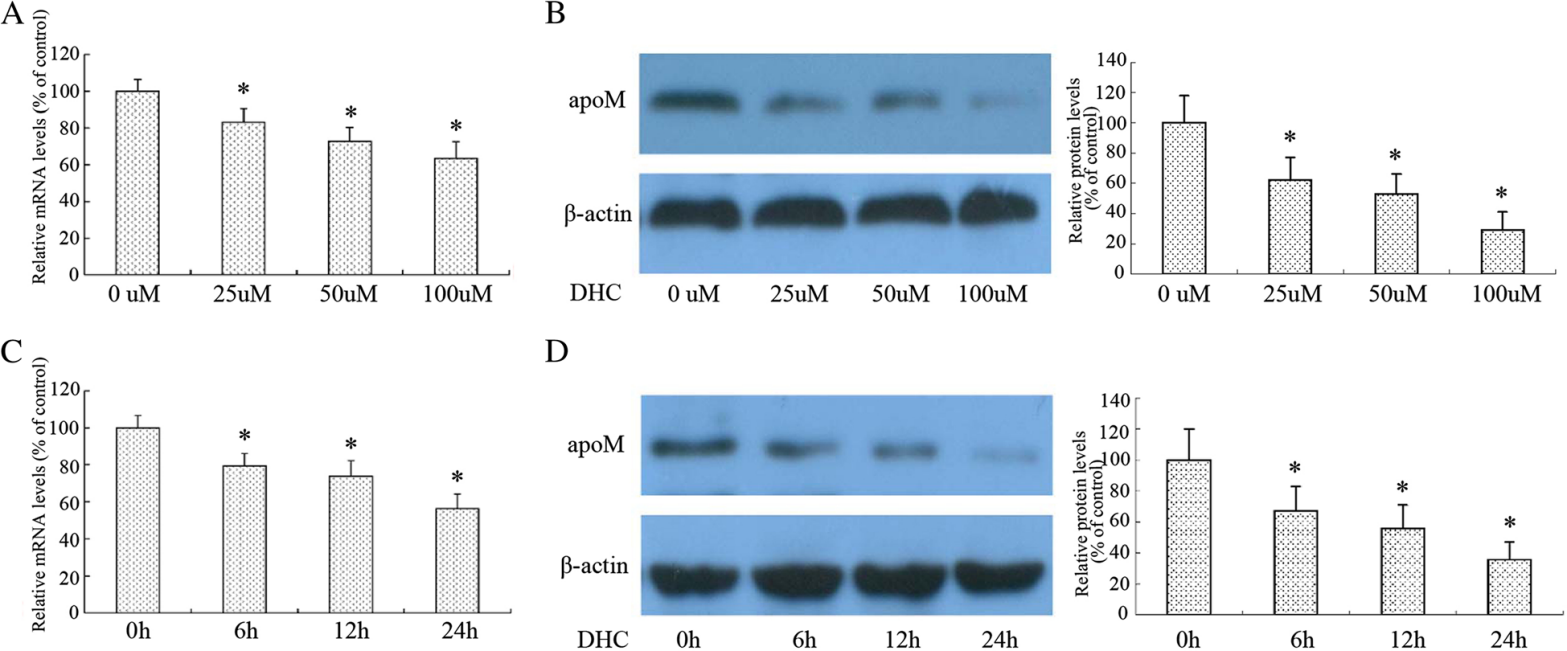
**Effect of DHC on apoM expression in HepG2 cells. (A** and **B)** HepG2 cells were divided into four groups and cultured in medium at 37°C containing 0 μM, 25 μM, 50 μM and 100 μM DHC for 24 h, respectively. ApoM transcript levels and protein levels were measured by real-time quantitative PCR and western blot analysis, respectively. **(C** and **D)** HepG2 cells were treated with 100 μM DHC for 0, 6, 12, and24 h, respectively. ApoM transcript levels and protein levels were measured by real-time quantitative PCR and western blot analysis, respectively. All results are presented as mean ± SD of three independent experiments, each performed in triplicate. *P < 0.05 vs. the control group.

ApoM is highly expressed in the liver and kidney in humans, mice, and pigs. In the liver, apoM is expressed in hepatocytes and mainly secreted into the plasma, where it becomes integrated in plasma lipoproteins. We first examined the effect of DHC on apoM expression in HepG2 cells by real-time quantitative PCR and Western blot assays. As shown, DHC obviously decreased apoM expression at both transcriptional levels and translational levels in a dose-dependent (Figure 1A and B) and time-dependent manner (Figure 1C and D).

### Foxa2 is involved in DHC-induced down-regulation of apoM in HepG2 cells

**Figure 2:**
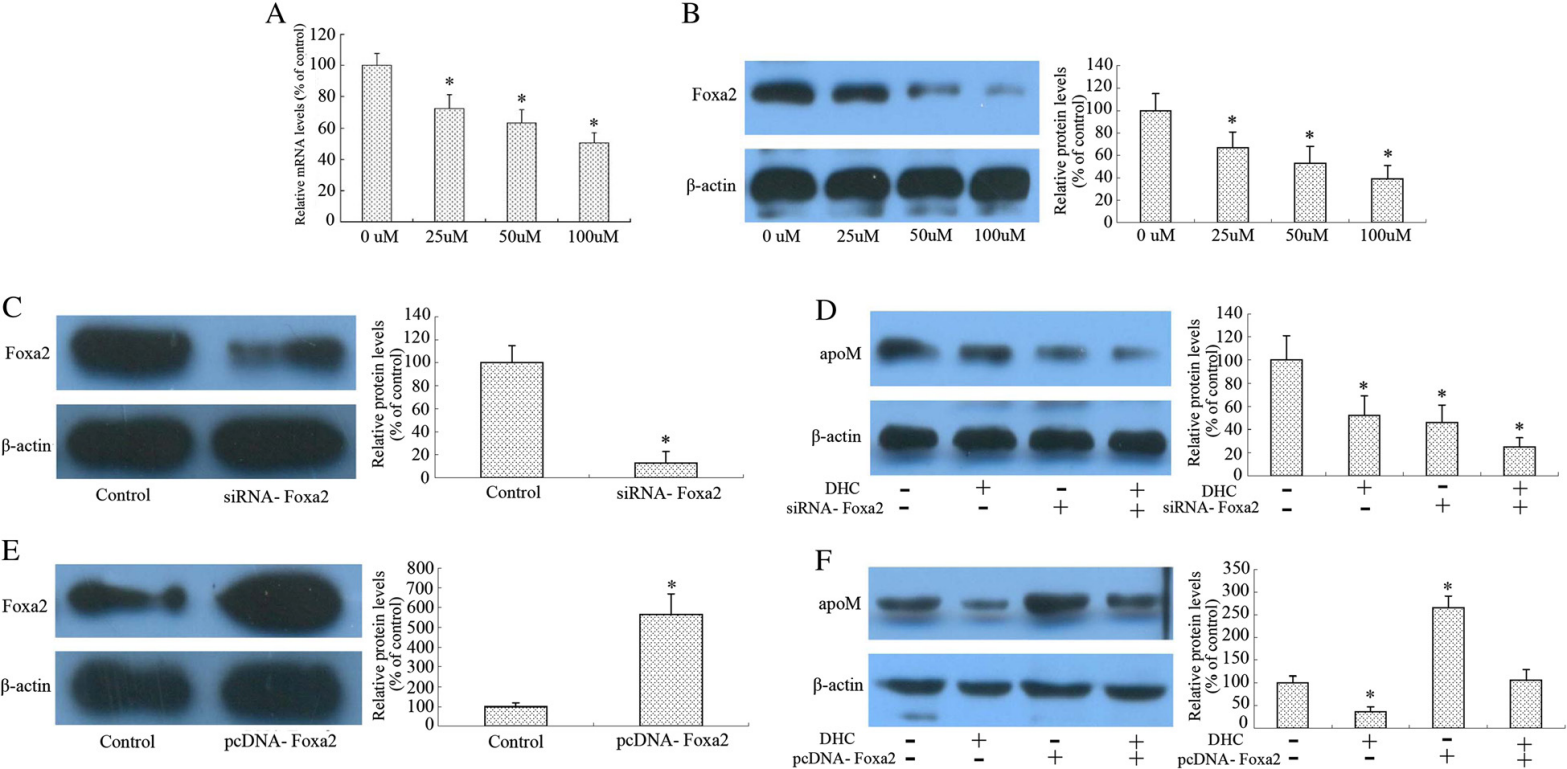
**Effect of DHC on Foxa2 and expression in HepG2 cells. (A** and **B)** HepG2 cells were divided into four groups and cultured in medium at 37°C containing 0 μM, 25 μM, 50 μM and 100 μM DHC for 24 h, respectively. Foxa2 transcript levels and protein levels were measured by real-time quantitative PCR and western blot analysis, respectively. **(C)** HepG2 cells were transfected with negative control or Foxa2 siRNA. And then protein samples were measured by western blot analysis. **(D)** HepG2 cells were transfected with Foxa2 or control siRNA and then incubated with 100 μM DHC for 24 h. And then protein samples were measured by western blot analysis. **(E)** HepG2 cells were transfected with pcDNA3.1-mock (control group) or pcDNA-Foxa2 (pcDNA group). And then protein samples were measured by western blot analysis. **(F)** HepG2 cells were transfected with pcDNA-Foxa2 or pcDNA-control and then incubated with 100 μM DHC for 24 h. Protein samples were measured by western blot analysis. All results are presented as mean ± SD of three independent experiments, each performed in triplicate. *P < 0.05 vs. the control group.

Previously researches revealed that apoM expression is directly regulated by Foxa2, which is a transcription factor involved in hepatic development via the regulation of glucose homeostasis in liver. We next explore whether Foxa2 expression can be affected by DHC in HepG2 cells by real-time quantitative PCR and western blot analysis. As shown, DHC obviously decreased Foxa2 mRNA and protein expression in a dose-dependent (Figure 2A and B). We then examined the effect of Foxa2 siRNA on the down-regulation of apoM which was induced by DHC. As shown in Figure 2C, In comparison to the control siRNA, treatment with siRNA targeting Foxa2 decreased Foxa2 protein expression by 87% in HepG2 cells. The down-regulation of apoM expression via DHC treatment was markedly accentated by Foxa2 siRNA treatment (Figure 2D). Next, we observed the effects of recombinant plasmids over-expressing Foxa2 (pcDNA-Foxa2) on apoM expression following DHC treatment in HepG2 cells. As shown in Figure 2E, treatment with pcDNA-Foxa2 increased Foxa2 protein expression by 565% in HepG2 cells. Suppression of apoM expression by DHC was markedly compensated by treatment with pcDNA- Foxa2 (Figure 2F).

### DHC down-regulates apoM expression through enhancing LXRα expression in HepG2 cells

**Figure 3:**
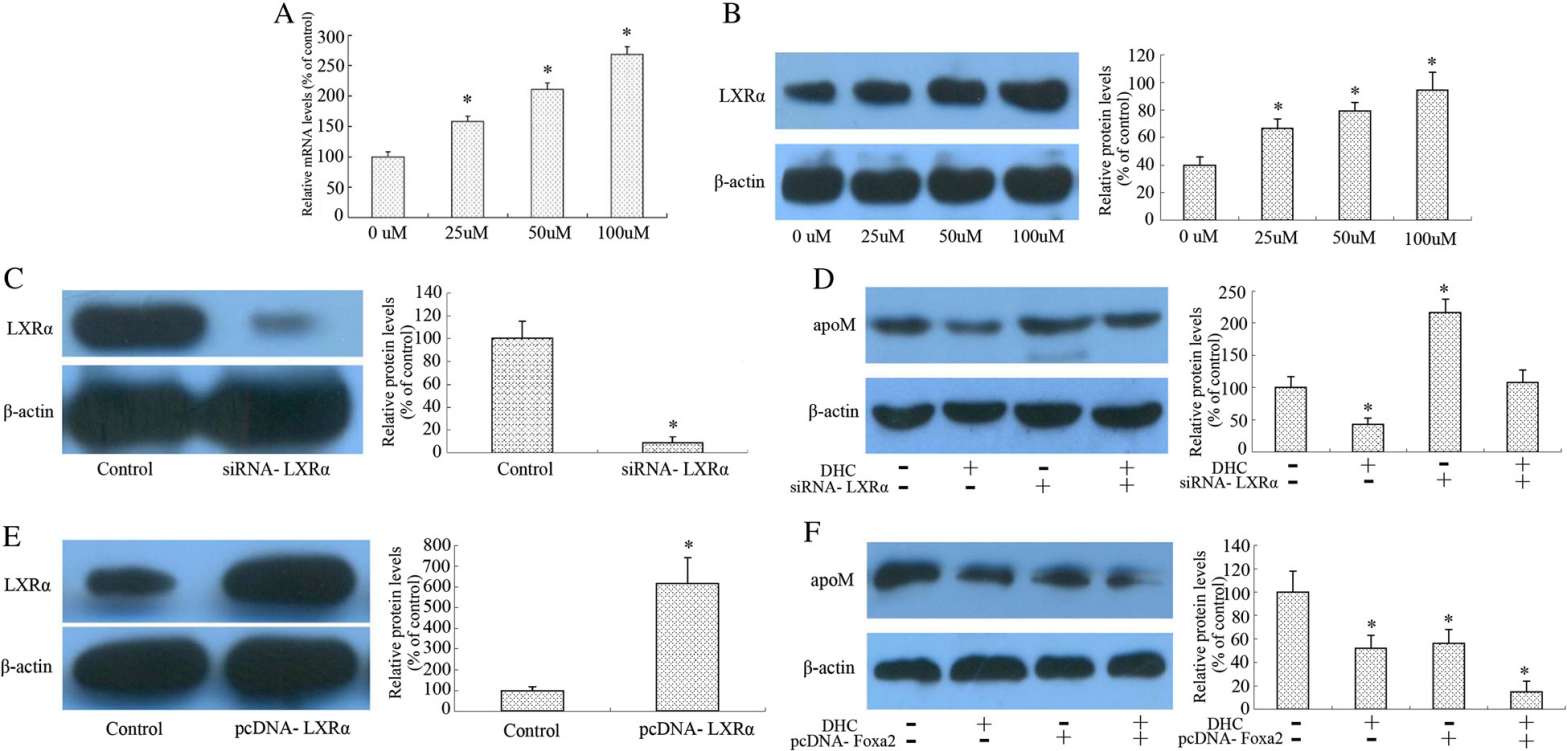
**Effect of DHC on LXRα and expression in HepG2 cells. (A** and **B)** HepG2 cells were divided into four groups and cultured in medium at 37uC containing 0 μM, 25 μM, 50 μM and 100 μM DHC for 24 h, respectively. LXRα transcript levels and protein levels were measured by real-time quantitative PCR and western blot analysis, respectively. **(C)** HepG2 cells were transfected with negative control or LXRα siRNA. And then protein samples were measured by western blot analysis. **(D)** HepG2 cells were transfected with LXRα or control siRNA and then incubated with 100 μM DHC for 24 h. And then protein samples were measured by western blot analysis. **(E)** HepG2 cells were transfected with pcDNA3.1-mock (control group) or pcDNA-LXRα(pcDNA group). And then protein samples were measured by western blot analysis. **(F)** HepG2 cells were transfected with pcDNA-LXRα or pcDNA-control and then incubated with 100 μM DHC for 24 h. Protein samples were were measured by western blot analysis. All results are presented as mean ± SD of three independent experiments, each performed in triplicate. *P < 0.05 vs. the control group.

The previous study has proved that apoM mRNA levels were significantly lower in the presence of LXR agonists in HepG2 cell. A similar reduction was found *in vivo*. We next investigated whether LXRα involved in DHC included down-regulation of apoM. For this purpose, we performed gene and protein expression of LXRα in HepG2 cells following treatment with DHC. As shown, DHC obviously increased LXRα mRNA and protein expression in a dose-dependent manner (Figure 3A and B). As shown in Figure 3C, in comparison to the control siRNA, treatment with siRNA targeting LXRα decreased LXRα protein expression by 91% in HepG2 cells. The down-regulation of apoM expression by DHC treatment was markedly abolished by LXRα siRNA treatment (Figure 3D). In addition, we observed the effects of recombinant plasmids over-expressing LXRα (pcDNA-LXRα) on apoM expression following DHC treatment in HepG2 cells. As shown in Figure 3E, treatment with pcDNA-LXRα increased LXRα protein expression by 617% in HepG2 cells. Suppression of apoM expression by DHC was markedly accentated by treatment with pcDNA-LXRα (Figure 3F).

### Effect of DHC on hepatic tissue apoM expression

**Figure 4:**
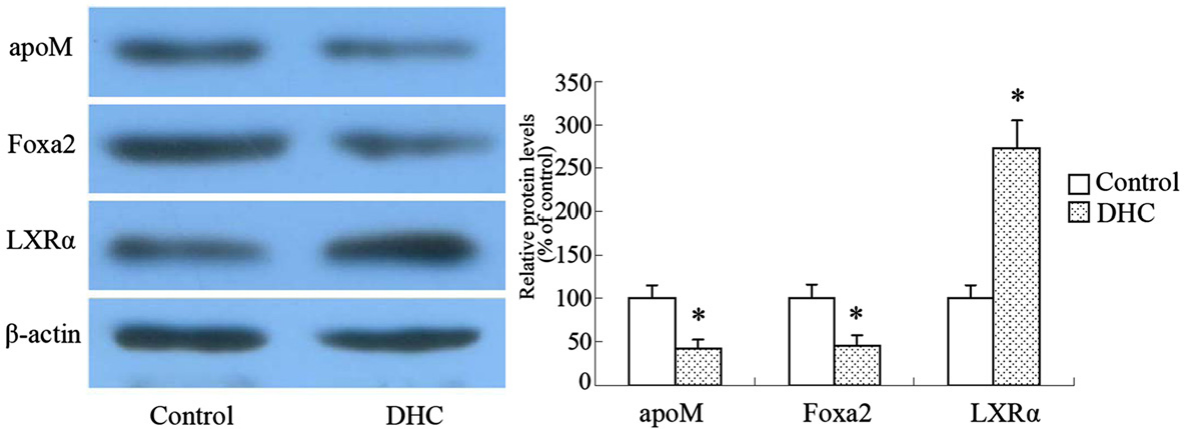
**Effect of DHC on hepatic apoM, Foxa2 and LXRα expression.** C57BL/6 Mice were randomized into the control group or the DHC group, and treated with either vehicle (cholesterol-free vegetable oil) or DHC (3.0 mg/kg body weight) daily by oral gavage for 1 week. The protein expression of apoM, Foxa2 and LXRα was measured by western blot. All results are presented as mean ± SD of three independent experiments, each performed in triplicate. *P < 0.05 vs. the control group.

The liver is the major organ responsible for the production and degradation of apoM-containing lipoproteins. Therefore, we next analyzed the effect of DHC on apoM, Foxa2 and LXRα expression in the liver of C57BL/6 Mice by western blot analyses. The control and DHC groups were treated with either vehicle (cholesterol-free vegetable oil) or with DHC (3.0 mg/kg body weight, dissolved in cholesterol-free vegetable oil) daily by oral gavage (0.2 mL per mouse) for one weeks and then protein levels in C57BL/6 Mice liver tissues were investigated by western blot. As shown (Figure 4), the DHC group had significantly lower expression of apoM and Foxa2 than the control group while the DHC group had higher expression change of LXRα as compared to the control group.

## Discussion

Capsaicinoids, including capsaicin and dihydrocapsaicin, are the major pungent constituents of ‘hot chilli peppers’ of the Capsicum genus. In addition to food additive uses in our diet, the other biological properties and medical applications of capsaicin make this compound very popular. Previous studies have demonstrated that capsaicin is able to stimulate the release of calcitonin gene-related peptide (CGRP) by activating transient receptor potential channel vanilloid type 1 (TRPV1) and therefore it has potential benefits for cardiovascular function [29]. Furthermore, capsaicinoids can also contribute to their beneficial effects on the cardiovascular system through their antioxidant properties [30,31]. Our group have recently shown that DHC can significantly decrease atherosclerotic plaque formation involving in a PPARγ/LXRα pathway in apoE−/− mice fed a high-fat/high-cholesterol diet [1]. In the present study, we demonstrated that apoM expression was regulated by DHC in HepG2 cells in a dose-dependent and time-dependent manner and that inhibition of apoM expression by DHC was mediated by Foxa2 and LXRα.

ApoM is a recently discovered apolipoprotein that is important for pre-β-HDL formation [32-35]. Overexpression of apoM in mice increases plasma HDL-cholesterol, and apoM-deficiency decreases HDL-cholesterol [32]. Moreover, apoM-enrichment of HDL augments the ability of HDL to mobilize cholesterol from macrophage-derived foam cells [32,36]. Here, we found that DHC could decrease apoM expression through inhibiting Foxa2 expression and enhancing LXRα expression in HepG2 cells. ApoM is predominantly associated with HDL (96% is bound to HDL), but only 5% of the plasma HDL particles contain an apoM molecule [36]. Although it has been proved that apoM-deficient led to the appearance of plasma HDL particles that were larger than those of normal mice,which impaired in their ability to transport cholesterol out of macrophages [24], the effect of apoM-deficient on HDL is limited since just 5% of the plasma HDL particles contain an apoM molecule. In addition, apoM is a negative acute response protein that decreases during inflammation. The level of apoM in mouse serum decreased following LPS administration and decrease of its expression in the liver occurs rapidly [37]. Inflammation could increase the risk for atherosclerosis including alterations in lipid and lipoprotein metabolism. Besides, HDL isolated during inflammaion is poorer at removing cholesterol from macrophages than normal HDL [38]. Zhang et al. showed that high glucose could inhibit apoM expression in HepG2 cells. Serum apoM concentrations and hepatic apoM mRNA levels were significantly reduced in the hyperglycemic rats, indicating that the low expression levels of apoM in these diabetic animals could be ascribed to hyperglycemia [20]. Similarly, plasma apoM is modestly reduced in patients with diabetes compared to controls [19]. These observations suggest that apoM is link to the development and progression of atherosclerosis and diabetes. In the present study, we found that DHC could decrease apoM expression by inhibiting Foxa2 expression and enhancing LXRα expression in HepG2 cells. The interesting thing was that our previous study revealed that DHC could significantly decrease atherosclerotic plaque formation through enhancing expression of PPARγ and LXRα in apoE−/− mice fed a high-fat/high-cholesterol diet [1]. Here, we demonstrated that DHC could enhance LXRα expression, which was consistent with our previous study. However, it is important to consider the inhibition of apoM expression by DHC when it was used as the therapeutic drug for atherosclerosis and diabetes. Thus, more experiments should be performed to prove DHC might be potentially used to treat the patients with atherosclerosis and diabetes.

Foxa2, a key regulator of hepatic lipid metabolism, which is transcriptionally active in the fasted state and induces expression of enzymes involved in fatty acid oxidation, ketogenesis, and VLDL secretion and bile acid metabolism, is regulated via insulin/PI3K/Akt-mediated phosphorylation at a single conserved threonine (Thr156) residue [39]. Previous studies have shown that apoM expression and plasma pre-β-HDL levels are decreased in obese mice due to inactivation of Foxa2 in the accompanying hyperinsulinemic state [23]. Treatment of wild-type mice and ob/ob mice with an adenovirus containing phosphorylation-defective Foxa2 not only improved glucose and lipid homeostasis but also increased hepatic apoM mRNA expression. In contrast, haplo-insufficient Foxa2+/−mice exhibited decreases in hepatic apoM expression and in plasma pre-β-HDL and HDL levels. A binding site for Foxa2 in the APOM promoter, at position −474, was identified [24]. In the present study, we showed that Foxa2 expression was significantly decreased by DHC treatment in HepG2 cells. The down-regulation of apoM expression via DHC treatment was markedly accentated by Foxa2 siRNA treatment. Moreover, the suppression of apoM expression by DHC was markedly compensated by treatment with pcDNA-Foxa2. These suggested that Foxa2 was involved in apoM expression regulation by DHC in HepG2 cells.

LXRα is expressed mainly in the liver, intestine, adipose tissue and macrophages [27]. LXRs are involved in the control of major metabolic pathways such as cholesterol homeostasis, lipogenesis, inflammation and innate immunity [26,40]. Zhang et al. demonstrated that TO901317 downregulates apoM expression in the liver. They showed that mice treated with 100 mg/kg/d of TO901317 had significantly lower apoM plasma levels as compared to control mice. In cultured HepG2 cells, TO901317 caused a downregulation of apoM expression,which was in line with the *in vivo* findings [27]. Thus, the agonist significantly decreases apoM mRNA expression *in vivo* and *in vitro*, indicating that APOM is another target gene of LXR. In the present study, we showed that the down-regulation of APOM expression via DHC treatment was markedly abolished by LXRα siRNA treatment. Moreover, the suppression of apoM expression by DHC was markedly accentated by treatment with pcDNA-LXRα, suggesting that LXRα is also involved in apoM expression regulation by DHC in HepG2 cells.

The liver is the major organ responsible for the production and degradation of apoM-containing lipoproteins [16]. We showed that oral gavage DHC can significantly decrease expression of apoM. Meanwhile, DHC can markedly decrease expression of Foxa2 than the control group while increase expression of LXRα as compared to the control group in the liver of C57BL/6 Mice. *In vivo* studies were consistent with the *in vitro* findings, demonstrating DHC down-regulates apoM expression through inhibiting Foxa2 expression and enhancing LXRα expression.

## Conclusion

Herein, we provided strong evidences that DHC down-regulates apoM expression through inhibiting Foxa2 expression and enhancing LXRα expression in HepG2 cells. This research provides a possibility to explain the molecular mechanism of DHC in down-regulation apoM expression in hepatic cells. Since DHC has multiple biological properties and medical applications, it still needs more experiment evidence to support DHC might be potentially used to treat the patients with atherosclerosis and diabetes.

## Competing interests

The authors declare that they have no competing interests.

## Authors’ contributions

JYZ, YCW and SFL participated in the assay of RT-PCR. JYZ, YWH, YRH, JJG and SFL participated in the assay of Western blotting. SGW and YHS participated in cell culture. QW and LZ participated in the design of study, performed the statistical analysis. JYZ and YWH drafted the manuscript. All authors read and approved the final manuscript.

## Authors’ information

Yan-Wei Hu is a co-first author.
